# Comparing the reasons for contraceptive discontinuation between parenting adolescents and young women in sub-Saharan Africa: a multilevel analysis

**DOI:** 10.1186/s12978-023-01660-6

**Published:** 2023-08-08

**Authors:** Sunday A. Adedini, Olusola A. Omisakin

**Affiliations:** 1 Department of Demography and Social Statistics, Faculty of Social Sciences, Federal University, Oye-Ekiti, Nigeria; 2 Demography and Population Studies Programme, Schools of Public Health and Social Sciences, University of the Witwatersrand, Johannesburg, South Africa; 3 Department of Sociology and Anthropology, Utah State University, Logan, UT USA; 4 Department of Demography and Social Statistics, Federal University, Birnin-Kebbi, Kebbi State Nigeria

**Keywords:** Contraception, Contraceptive discontinuation, Parenting adolescents, Parenting young women, Sub-Saharan Africa

## Abstract

**Background:**

Adolescent sexual and reproductive health remains a major public health and development issue of global importance. Given that adolescents and young people are heterogenous groups in terms of many characteristics, this study expands the literature by comparing the reasons for contraceptive discontinuation between parenting adolescents (aged 15–19) and parenting young women (aged 20–24) in sub-Saharan Africa (SSA).

**Methods:**

Data for the study came from Demographic and Health Surveys of 22 SSA countries. The outcome variable was reasons for discontinuation. We performed multilevel binary logistic regression on analytic samples comprising 1485 parenting adolescents and 10,287 parenting young women across the selected SSA countries.

**Results:**

Findings show that the proportion of respondents who used modern contraceptives was lower among parenting adolescents (35%) relative to their 20–24-year-old counterparts (43%). Higher percentages of parenting adolescents than young women discontinued contraceptives because of reasons such as pregnancy or method failure (i.e., 9.9% and 8.17% accordingly), husband disapproval, access or availability issues, wanting more effective methods, and inconvenience in using methods. The multilevel analysis further highlighted disparities between parenting adolescents and parenting young women who discontinued contraceptives. For instance, parenting young women had 30% lower odds of discontinuing contraceptives due to pregnancy or method failure than parenting adolescents.

**Conclusion:**

The study established disparities in the reasons for contraceptive discontinuation between parenting adolescents and parenting young women, with adolescents demonstrating greater vulnerabilities and higher risks. Considerable attention must be given to parenting adolescents in the efforts to achieve equity goals such as the Sustainable Development Goals and universal health coverage in SSA.

## Introduction

Adolescent girls continue to face great challenges in accessing sexual and reproductive health services in developing countries [1]. Adolescents aged 15–19 are a high-risk group for poor maternal health outcomes [2]. The global adolescent fertility rate is high at 49 births per 1000 girls aged 15–19 [3]. The high rate of adolescent pregnancy and fertility is largely due to limited access to information about sexual and reproductive health, poor access to reproductive health services, cultural beliefs, sexual violence, early and forced marriage, and declining age at menarche, amongst others [4–6]. Sub-Saharan Africa’s adolescent fertility rate of 99.6 (per 1000 women aged 15–19 years) is among the highest globally, with the rate as high as 175 and 152 births in Niger and Mali, respectively [7]. Evidence shows that nearly half of adolescent pregnancies in developing countries are unintended [8].

The focus of every family planning programme is to address the problem of unmet need for modern contraception and unintended pregnancy. While the risk of unintended pregnancy can be reduced among women through the appropriate use of contraception, adolescent girls generally have low contraceptive prevalence compared to the older age groups [9]. Meeting the adolescents’ needs for reproductive health services is an important health and development goal. However, adolescent girls face unique challenges and barriers to accessing sexual and reproductive health services, including health workers’ bias, lack or low availability of adolescent-and-youth-friendly reproductive health services, discriminating social norms and cultural and religious beliefs, as well as poor socioeconomic status [10–13].

Contraceptive discontinuation particularly among adolescent girls poses a great challenge to the attainment of many national and international goals such as Family Planning (FP) 2020 and FP 2030. Discontinuation of contraceptive use is a major concern among adolescents and young people. In a previous study, at least one episode of contraceptive discontinuation was reported among most of the adolescents and young women studied in Burkina Faso, Mali, and Niger [14]. Ouédraogo et al. established a discontinuation rate of 68.7% among the respondents. Some of the reasons for contraceptive discontinuations established in previous studies include dissatisfaction, health concerns or side effects, fear of becoming infertile, perceived dangers about continuous and prolonged use, as well as socio-economic characteristics [14, 15].

Long-acting reversible contraception, including implants and intra-uterine devices (IUDs), is recommended for adolescents and young women, albeit early discontinuation of these methods is widely reported [12, 16]. For instance, a study by Cohen et al. [11], which examined the predictors of discontinuing long-acting reversible contraceptive use among adolescents and young people established side effects such as pain (for IUD) and heavy bleeding (for implant) as reasons for discontinuation. Other studies have reported reasons for contraceptive discontinuation, including mood change, gender-based violence, method failure, peer pressure, history of sexually transmitted infection, cost of contraceptive commodities or services, bone density changes, and failure relating to the health system [12, 16, 17]. Although adolescents and young women report experiencing side effects from using contraception, evidence suggests that they rarely discuss this with their providers; rather they source information from the internet or peers [12].

Blanc et al. [10] did a comparative analysis of patterns of contraceptive use and discontinuation between adolescents and adult women and found that a higher proportion of adolescent girls than older women reported discontinuation of contraceptive use within a year of initiating methods. A recent study compared younger (15–24-year-old) versus older (25–49-year-old) women's contraceptive and abortion practices and reasons for discontinuing contraception [1] and found a higher discontinuation rate among younger compared to older women, with infrequent sex being the major reason for discontinuation. The literature largely assumes that adolescent girls aged 15–19 and young women aged 20–24 are homogenous groups. However, a growing body of literature has given recognition to a new life stage during the early 20 s due to longer time in education and before marriage and parenthood, thus making this group of young people uniquely different from older adolescents [18–20]. For instance, in the works of Arnett et al. [21] and Gilmore [22], young people in their early 20 s up to mid-20 s are considered emerging adults whose demographics are different from those of people younger than age 20. A recent study by Adedini et al. [23] established that young girls who married early at adolescent age and young women who married at a later age are significantly different in terms of many socio-economic characteristics (including educational attainment, occupation or employment status, media exposure, wealth status, etc.), thus affirming that older adolescents (15–19) and young people (20–24) are different in very many ways. To this end, the present study did a comparative analysis of reasons for discontinuing contraceptive use between parenting adolescent girls (15–19-year-old) and parenting young women (20–24-year-old) in sub-Saharan Africa.

## Data and methods

### Study designs and participants

We used cross-sectional datasets derived from the Integrated Public Use Microdata Series (IPUMS) harmonized Demographic and Health Surveys (DHS) for multiple countries from 2010 to 2018. Throughout sub-Saharan Africa and other developing countries, the DHS has been the principal source of comparable nationally representative data and addresses essential topical areas including individual and household socioeconomic conditions, birth history, maternal and child health, contraceptive use patterns, and health biomarkers. In addition, the integrated DHS has data files for a variety of units of analysis, such as births, couples, individual women, children, men, and household members. Individual recode file was used for this study.

In this study, we used DHS datasets from women living in 22 countries in sub-Saharan Africa: Angola (2015), Benin (2011), Burkina Faso (2010), Burundi (2010), Ethiopia (2016), Ghana (2014), Guinea (2018), Kenya (2014), Lesotho (2014), Malawi (2016), Mali (2012), Mozambique (2011), Namibia (2013), Niger (2012), Nigeria (2018), Rwanda (2014), Senegal (2017), South Africa (2016), Tanzania (2015), Uganda (2016), Zambia (2018), and Zimbabwe (2015). The DHS design and implementation procedures for each selected country have been published and are accessible via the DHS program website (dhsprogram.com).

The inclusion of countries and respondents in the current study was determined by a few criteria. First, we considered the most recent DHS for all the countries in sub-Saharan Africa. Countries were included if questions about contraceptive use and contraceptive discontinuation were asked. However, if both questions were not asked in a country’s most recent survey, the previous survey was chosen. This step led to the selection of 22 countries across sub-Saharan Africa. Since our goal was to assess reasons for contraceptive discontinuation, our second criterion involved selecting women who had discontinued contraceptive use within the last five years preceding the DHS data collection. Next, we restricted the sample to only parenting adolescents and parenting young women. We defined parenting adolescents as women aged 15–19 who had given birth to at least one child and parenting young women as those aged 20–24 who had given birth to at least one child. Finally, cases with missing data on any of the study variables were excluded, as there are only a few missing cases comprising 513 observations (4.29%). Thus, our weighted analytic samples comprised 1485 adolescents and 10,287 young women across the selected sub-Saharan African countries. In Table 1, we present the distribution of the study’s sample by country and for each group of women.

**Table 1:** Eligible study participants in selected sub-Sahara African countries

Country	Parenting adolescents (n)	Young women (n)	Weighted total
Angola (2015)	58	238	296
Burundi (2010)	12	186	197
Benin (2011)	17	164	181
Ethiopia (2016)	75	578	653
Ghana (2014)	33	163	196
Guinea (2018)	63	168	231
Kenya (2014)	78	683	761
Lesotho (2014)	14	187	201
Malawi (2016)	227	1922	2148
Mali (2012)	25	142	167
Mozambique (2011)	67	247	314
Namibia (2013)	73	454	527
Niger (2012)	54	327	381
Nigeria (2018)	42	305	347
Rwanda (2014)	18	341	359
Senegal (2017)	47	316	363
South Africa (2016)	28	250	277
Zimbabwe (2015)	85	653	738
Uganda (2016)	190	1234	1424
Tanzania (2015)	109	625	733
Burkina Faso (2010)	23	244	266
Zambia (2018)	149	860	1009
Weighted Total	1485	10,287	11,772

## Measures

### Outcome variables

The outcome variables in our study are reasons for discontinuation. During the DHS surveys, respondents identified more than 30 possible reasons for discontinuing the last contraceptive method they had used in the five years before the surveys. But after selecting the sample for the current study, we identified 12 specific reasons reported by parenting adolescents and young women for their last contraceptive discontinuation: became pregnant/method failed; wanted to get pregnant; husband disapproved; side effects or health concerns; access or availability issues; wanted more effective method; inconvenient to use; infrequent sexual activity, or husband away or ill; cost of method; fatalistic; difficulty getting pregnant, menopause or amenorrhea; marital dissolution/divorced or widowed. In our study, we treated each response item as a distinct outcome by obtaining dummy variable for each item, allowing us to look deeper into the reasons of discontinuing contraceptives between the two age groups of women.

### Other measures

We included measures of demographic characteristics and socioeconomic conditions as covariates in our study. These include area of residence (urban = 1 or rural = 2), marital status (never married = 1, ever married/cohabiting = 2), total children ever born (one = 1 or two/more = 2), wealth index (poorest = 1, poorer = 2, middle = 3, richer = 4, or richest = 5), highest level of education (no education = 1, primary = 2, or secondary/ higher = 3), religion (Christianity = 1 or non-Christians = 2), type of family (monogamous = 1, polygynous = 2, or not currently in a union = 3) and selected countries. Occupation was categorized as formal employment = 1 (professional, technical, managerial, clerical, sales, services, or skilled manual workers), agricultural employment = 2, unskilled manual workers = 3 (household and domestic workers, or unspecified unskilled manual worker), and unemployed = 4.

In addition, the DHS surveys employed principal component analyses to categorize women into five levels of wealth index (poorest, poorer, middle, richer, and richest) according to several indicators of women's household wealth, such as access to drinking water, electricity, land or livestock, and toilet facilities. Type of family was evaluated based on number of other wives as self-reported by the respondents. Thus, we categorized family type as monogamous if there were no other wives, and polygynous provided there were one or more other wives. The rest of the respondents who were not currently in a union were grouped in a third category.

### Statistical analysis

Analyses were conducted by utilizing Stata 16.1 [24]. We weighted the data for all analyses so that we could ensure that estimates are accurate and representative of parenting adolescents and young women. As a first step, we obtained percentage distributions of parenting adolescents and young women’s by background characteristics and performed the chi-square test of association to assess differences between the two groups. In our second step, we obtained percentage distributions to compare and contrast parenting adolescents' reasons for discontinuing contraceptives with those of young women.

We viewed the data as having two levels of nesting. All types of measures related to individual respondents (micro units) are nested within each selected country (macro units). Consequently, the next phase in our analysis involved estimating a series of multilevel models to account for disparities in parenting adolescents and young women’s reasons for discontinuing contraceptives as well as factors that contribute to the disparities. While generalized linear models can also be used, these models suffer from some fundamental shortcomings when applied to nested data. For instance, the use of generalized linear model assumes independence of observation, ignoring the group differences between subjects. Our choice of statistical method is further justified in that multilevel statistical models have been demonstrated to provide estimation that is valid and reliable if at least five observations are available for the group [25]. Thus, by utilizing a multilevel modelling, our study provides a more thorough explanation of disparities in reasons for contraceptive discontinuation than the traditional regression models.

We estimated fixed effects using two-level binary logistic regression models. To compare the odds ratios of reasons for contraceptive discontinuation between parenting adolescents and young women, we developed baseline models that included the outcome variables and the two age groups of women. Then, we estimated larger models that combined the baseline model and the covariates to show disparities in the odds ratios of reasons for contraceptive discontinuation between the two age groups when all covariates were held constant.

## Results

Table 2 provides the background characteristics of the respondents. For each group of parenting adolescents and parenting young women in selected countries in sub-Saharan Africa, two-third of the respondents lived in rural areas. The distributions for each group of respondents are also similar in the sense that highest proportions were ever married or cohabiting, unemployed, attained primary education, practiced Christian religion, and belonged to monogamous family type. Nonetheless, parenting adolescents and young women exhibited some dissimilar characteristics. For instance, 24% of parenting adolescents were not in a union as compared to 12% of the young women. Most of the parenting adolescents (76%) were primiparous, whereas 60% of the young women were multiparous. A large proportion were unemployed: 45% of parenting adolescents and 36% of young women. Approximately 60% of parenting adolescents belonged to the poorest, poorer, or middle households, as compared to 56% of young women. Except for area of residence, all the variables were significantly associated with disparities between distributions of women 15–19 and 20–24 years.

**Table 2:** Percentage distribution of parenting adolescents and young women’s sociodemographic characteristics

Variables	Parenting adolescents	Parenting young women	Chi-square (p-value)
Place of residence			0.014 (0.907)
Urban	34.1	34.2	
Rural	65.9	65.8	
Current marital status			184.6 (0.000)
Never married	24.2	11.5	
Cohabiting or ever married	75.8	88.5	
Total children ever born			675.7 (0.000)
One	76.2	40.3	
Two or more	23.8	59.7	
M (SD)	1.26 (0.49)	1.83 (0.85)	
Occupation			43.04 (0.000)
Formal employment	20.9	24.7	
Agricultural employment	24.5	28.3	
Unskilled manual worker and other	9.6	10.7	
Unemployed	45.0	36.3	
Wealth index			25.9 (0.000)
Poorest	20.8	16.4	
Poorer	21.6	20.5	
Middle	18.3	19.2	
Richer	22.1	22.9	
Richest	17.1	20.9	
Highest educational level			43.4 (0.000)
No education	11.3	12.7	
Primary	56.1	47.0	
Secondary or higher	32.6	40.3	
Religion			4.6 (0.031)
Christianity	67.8	70.5	
Non-Christians	32.2	29.5	
Type of family			122.9 (0.000)
Monogamous	60.0	70.8	
Polygynous	5.6	7.7	
Not currently in a union	34.4	21.5	
Total (N)	1,485	10,287	

In Table 3, we present the reasons for contraceptive discontinuation for each group of parenting adolescents and young women. Overall, slightly higher percentages of parenting adolescents than young women discontinued contraceptives because of reasons such as pregnancy or method failure (i.e., 9.9% and 8.2% accordingly), husband disapproval, access or availability issues, wanting more effective methods, inconvenience in using methods, infrequent sexual activity, cost of method, fatalistic beliefs, and marital dissolutions. Percentages of parenting adolescents who discontinued contraceptives were lower than among young women with respect to other reasons including intention to become pregnant (i.e., 22.7% and 31.4% accordingly), side effects or health concerns, and difficulty getting pregnant. Across the selected countries, percentages of parenting adolescents and young women who discontinued contraceptives varied. For instance, Namibia had the largest percentage (36.7%) of parenting adolescents who discontinued contraceptives because of pregnancy or method failure, but Ghana had the highest percentage among young women (23.6%). The results show that the highest percentage of parenting adolescents in South Africa (37.1%) and highest proportion of young women in Uganda (36.2%) discontinued contraceptives because of side effects or health concerns.

**Table 3:** Percentage distributions of parenting adolescents and young women by type of reasons for contraceptive discontinuation in selected sub-Saharan African countries

Countries	Became pregnant/method failed	Wanted to get pregnant	Husband disapproved	Side effects or health concerns	Access or availability issues	Wanted more effective method	Inconvenient to use	Infrequent sexual activity	Cost of method	Fatalistic	Difficulty getting pregnant	Marital dissolution
Parenting adolescents												
Angola	10.2	7.6	10.1	0	0.7	0.4	11.9	13.2	0	6.5	0	0
Burundi	0	8.2	0	0	0	0	26.6	23.9	0	0	0	5.6
Benin	0	29.3	12.9	5.1	4.6	10.0	0	0	0	7.3	0	4.7
Ethiopia	8.5	55.1	2.2	5.6	9.4	0.8	3.5	3.2	0	0	0	4.6
Ghana	34.3	20.2	0	10.3	5.1	2.0	6.6	20.4	1.1	0	0	0
Guinea	2.3	34.6	5.4	31.1	0	5.5	9.9	4.7	0	0	0	0
Kenya	18.9	27.4	6.4	20.3	0.8	13.0	0.2	8.8	0	0	0	1.3
Lesotho	9.0	13.1	10.3	0	9.5	22.2	0	26.6	0	0	0	0
Malawi	3.2	19.2	4.0	17.1	7.3	18.4	1.8	14.7	0.8	0.2	0	5.2
Mali	5.7	30.8	3.9	19.1	1.8	8.9	8.0	3.3	0	3.7	2.2	0
Mozambique	10.4	26.3	11.2	25.8	3.6	2.9	0.8	5.0	0	0	0.7	0
Namibia	36.7	15.8	4.7	16.8	8.8	3.2	4.4	3.1	0	1.2	0	0.8
Niger	4.1	35.8	2.1	3.5	0	7.0	4.2	14.8	0	1.6	0	5.4
Nigeria	22.8	17.8	1.4	9.8	8.2	4.7	9.1	13.4	0	0	0	0
Rwanda	0	11.7	0	45.1	0	17.5	0	0	0	6.7	0	12.1
Senegal	0	29.4	21.8	0	1.5	3.1	5.2	4.9	0	0	0	0
South Africa	13.7	8.6	4.8	37.1	9.5	7.1	4.8	0.7	0	0	0	1.4
Zimbabwe	2.6	10.4	2.8	27.3	3.2	12.2	4.3	16.0	2.1	0	0	7.8
Uganda	9.6	20.1	10.3	30.8	1.3	5.8	5.3	8.1	4.4	0	0	1.2
Tanzania	10.6	27.4	4.8	33.1	0.9	8.6	0	10.3	1.8	0	0	0.1
Burkina Faso	23.0	32.9	0	12.4	13.9	0	3.5	5.7	8.8	0	0	0
Zambia	7.3	16.2	6.9	28.2	5.6	7.2	2.3	9.9	0.3	0	0	0.7
Total	9.9	22.7	6.2	20.5	4.2	8.2	3.9	9.8	1.1	0.6	0.1	2.3
Parenting young women												
Angola	7.5	15.8	6.9	0	0.6	7.1	9.8	12.9	3.6	0.6	0	1.5
Burundi	9.1	34.3	7.0	0	2.7	2.5	5.8	2.1	0.0	1.2	0	3.3
Benin	12.5	37.4	4.6	7.9	0.2	10.4	4.3	0	1.6	3.8	0	0.6
Ethiopia	1.6	55.6	0.6	14.7	4.3	6.6	4.8	5.5	0	0.7	0	1.8
Ghana	23.6	24.0	5.3	22.7	0.5	3.8	6.5	7.0	2.1	0.0	0	0.5
Guinea	2.4	35.7	7.1	17.5	1.7	3.2	8.7	11.6	2.6	1.4	0	0
Kenya	12.9	29.0	1.6	31.7	1.4	6.4	2.2	8.0	1.4	0.1	0	1.8
Lesotho	18.7	13.0	7.5	19.4	8.9	7.5	2.8	10.3	0	0	0	0
Malawi	2.8	26.2	3.7	27.9	6.3	9.8	3.2	8.9	0.6	0.6	0.3	3.2
Mali	7.5	34.9	11.5	16.7	1.9	5.4	2.9	4.6	1.2	0.5	1.5	0
Mozambique	5.4	31.9	8.6	20.3	3.9	3.3	1.6	6.9	0.5	0.6	0	1.6
Namibia	22.8	20.2	4.1	27.8	6.2	6.4	2.7	4.3	0	0	0	1.1
Niger	1.4	53.1	2.8	4.1	1.6	3.9	0.5	15.0	1.5	1.5	0.9	1.3
Nigeria	14.6	36.7	5.8	12.8	1.5	6.6	6.2	8.0	0.9	0.2	0	0.8
Rwanda	7.9	30.2	0.6	33.7	0.9	10.6	1.6	11.5	0	0.2	0	0.9
Senegal	2.2	34.4	11.8	0	0.2	4.2	2.0	7.9	0	0.7	0	2.5
South Africa	8.1	13.4	3.4	24.9	2.9	10.9	7.3	7.5	1.8	0.6	0	0
Zimbabwe	14.1	31.9	3.8	21.1	3.2	10.1	3.2	5.7	1.0	0	0	2.2
Uganda	7.3	27.2	6.4	36.2	0.9	4.1	2.5	8.0	1.1	0.1	0.1	1.6
Tanzania	10.3	40.3	2.9	28.6	2.0	6.1	0.6	4.0	1.2	0	0	1.7
Burkina Faso	7.5	49.7	4.0	22.8	1.7	3.4	1.1	2.4	3.7	0	0	0.4
Zambia	7.2	29.1	5.2	30.4	3.9	7.8	2.3	8.8	0	0.2	0	0.8
Total	8.2	31.4	4.5	23.9	3.2	7.0	3.1	7.6	0.9	0.4	0.1	1.7

Next, we estimated multilevel binary logistic regression models to compare reasons why parenting adolescents and young women stopped using contraceptives. Regression analysis was performed with respect to every reason identified among respondents for contraceptive discontinuation, but we only present some of the relevant results as shown in Table 4. The best-fitting model, which is typically the model with lower log-likelihood statistics, was mainly interpreted for each reason for contraceptive discontinuation. One of the reasons for discontinuing contraceptives was becoming pregnant or method failure. Results in Table 4 show that there were disparities between parenting adolescents who discontinued contraceptives owing to pregnancy or method failure and parenting young women. Young women had a 30% lower adjusted odds ratio (AOR) of discontinuing contraceptives due to pregnancy or method failure than parenting adolescents. Factors including current marital status, parity, level of education, and the country of residence all played a role in the found disparity. More specifically, respondents who cohabited or were ever married (AOR = 0.69; p-value < 0.001), had two or more children (AOR = 1.59; p-value < 0.001), and attained a secondary or higher level of education (AOR = 1.92; p-value < 0.001) significantly contributed to the decreased likelihood that young women would discontinue contraceptives due to pregnancy or method failure compared to parenting adolescents. Additionally, AORs of discontinuing contraceptives due to pregnancy or method failure were higher in many selected countries (Benin, Ghana, Kenya, Lesotho, Namibia, Nigeria, Zimbabwe, Tanzania, and Burkina Faso) and lower in other countries (Ethiopia, Malawi, and Senegal) as compared to Angola, contributing to the decreased likelihood of discontinuing contraceptives among young women compared to parenting adolescents.

**Table 4:** Multilevel binary logistic regression models

Fixed effects	Became pregnant/method failed			Husband disapproved			Wanted a more effective method		
	Model 1	Model 2	Model 3	Model 4	Model 5	Model 6	Model 7	Model 8	Model 9
	OR (SE)	OR (SE)	OR (SE)	OR (SE)	OR (SE)	OR (SE)	OR (SE)	OR (SE)	OR (SE)
Intercept	0.10 (0.02)***	0.10 (0.02)***	0.07 (0.02)***	0.06 (0.01)***	0.10 (0.02)***	0.10 (0.03)***	0.08 (0.01)***	0.07 (0.02)***	0.07 (0.23)***
*Individual characteristics*									
Age group									
Parenting adolescents (age 15–19)	1			1			1		
Parenting young women (age 20–24)	0.80 (0.08)*	0.79 (0.08)*	0.70 (0.07)**	0.77 (0.09)*	0.78 (0.09)*	0.78 (0.10)*	0.79 (0.08)*	0.78 (0.08)*	0.81 (0.09)
Place of residence									
Urban			1			1			1
Rural			0.97 (0.09)			1.37 (0.18)*			1.02 (0.11)
Current marital status									
Never married			1			1			1
Cohabiting or ever married			0.69 (0.07)***			1.53 (0.26)*			1.30 (0.17)*
Total children ever born									
One			1			1			1
Two or more			1.59 (0.12)***			1.04 (0.10)			0.76 (0.06)**
Occupation									
Formal employment			1			1			1
Agricultural employment			1.01 (0.11)			0.91 (0.13)			0.90 (0.11)
Unskilled manual worker and other			1.01 (0.13)			0.87 (0.16)			0.85 (0.12)
Unemployed			0.93 (0.09)			1.06 (0.13)			0.83 (0.08)
Wealth index									
Poorest			1			1			1
Poorer			1.07 (0.12)			0.78 (0.10)			1.07 (0.13)
Middle			0.88 (0.11)			0.75 (0.11)*			1.03 (0.13)
Richer			1.04 (0.13)			0.70 (0.11)*			0.97 (0.13)
Richest			1.05 (0.15)			0.71 (0.13)			1.40 (0.21)*
Highest educational level									
No education			1			1			1
Primary			1.52 (0.25)*			1.13 (0.18)			0.99 (0.15)
Secondary or higher			1.92 (0.32)***			0.92 (0.16)			1.01 (0.16)
Religion									
Christianity			1			1			1
Non-Christians			0.81 (0.10)			0.87 (0.13)			0.61 (0.09)***
*Country-level characteristic*									
Selected SSA countries									
Angola		1			1			1	
Burundi		1.12 (0.37)	1.73 (0.61)		0.89 (0.32)	0.60 (0.24)		0.41 (0.22)	0.40 (0.22)
Benin		1.51 (0.48)	2.33 (0.76)*		0.71 (0.28)	0.55 (0.23)		1.95 (0.68)	2.0 (0.72)
Ethiopia		0.29 (0.10)***	0.47 (0.16)*		0.10 (0.05)***	0.07 (0.03)***		1.07 (0.32)	1.10 (0.35)
Ghana		3.95 (1.07)***	4.75 (1.31)***		0.57 (0.23)	0.45 (0.19)		0.60 (0.28)	0.61 (0.28)
Guinea		0.27 (0.13)**	0.46 (0.23)		0.86 (0.30)	0.77 (0.29)		0.64 (0.27)	0.90 (0.40)
Kenya		1.83 (0.44)*	2.13 (0.52)**		0.27 (0.09)***	0.20 (0.07)***		1.28 (0.37)	1.29 (0.38)
Lesotho		2.61 (0.74)**	2.98 (0.87)***		1.06 (0.36)	0.90 (0.32)		1.59 (0.56)	1.46 (0.53)
Malawi		0.35 (0.09)***	0.44 (0.11)**		0.49 (0.12)**	0.31 (0.08)***		2.01 (0.52)**	2.11 (0.58)**
Mali		0.91 (0.33)	1.53 (0.60)		1.44 (0.48)	1.20 (0.45)		1.04 (0.43)	1.61 (0.71)
Mozambique		0.79 (0.25)	0.92 (0.29)		1.23 (0.36)	0.99 (0.30)		0.54 (0.22)	0.55 (0.23)
Namibia		3.84 (0.91)***	3.87 (0.95)***		0.54 (0.17)*	0.52 (0.17)*		1.06 (0.33)	1.20 (0.38)
Niger		0.21 (0.09)***	0.40 (0.19)		0.34 (0.13)**	0.26 (0.11)**		0.75 (0.27)	1.19 (0.47)
Nigeria		2.16 (0.56)**	2.51 (0.68)**		0.70 (0.23)	0.59 (0.20)		1.14 (0.38)	1.21 (0.41)
Rwanda		0.97 (0.28)	1.23 (0.38)		0.07 (0.05)***	0.05 (0.04)***		2.09 (0.63)*	2.0 (0.63)*
Senegal		0.23 (0.10)**	0.37 (0.17)*		1.88 (0.51)*	1.60 (0.51)		0.70 (0.26)	1.10 (0.43)
South Africa		1.11 (0.34)	1.28 (0.43)		0.47 (0.18)	0.56 (0.25)		1.98 (0.63)*	3.71 (1.32)***
Zimbabwe		1.71 (0.41)*	1.95 (0.48)**		0.48 (0.14)*	0.33 (0.10)***		1.93 (0.54)*	1.94 (0.55)*
Uganda		0.96 (0.23)	1.11 (0.27)		0.93 (0.23)	0.64 (0.17)		0.76 (0.21)	0.75 (0.22)
Tanzania		1.34 (0.33)	2.04 (0.57)*		0.41 (0.12)**	0.35 (0.12)**		1.16 (0.34)	1.81 (0.60)
Burkina Faso		1.14 (0.35)	1.98 (0.64)*		0.48 (0.19)	0.40 (0.17)*		0.54 (0.23)	0.63 (0.28)
Zambia		0.91 (0.22)	1.03 (0.26)		0.72 (0.19)	0.50 (0.14)*		1.39 (0.38)	1.42 (0.40)
Random effects									
Intercept variance	0.62 (0.21)	3.38e-34 (1.26e-18)	9.47e-34 (2.19e-18)	0.38 (0.14)	7.95e-34 (2.45e-18)	4.37e-33 (6.07e-18)	0.15 (0.06)	6.76e-34 (1.90e-18)	1.93e-33 (3.10e-18)
Model Fit									
-2log-likelihood	6,374.21	6,287.27	6,220.75	4,381.43	4,312.49	4,266.43	5,972.23	5,914.90	5,865.36

Multilevel binary logistic regression models to compare reasons for discontinuing contraceptives between parenting adolescents and young women in selected sub-Sahara African countries

OR: Odds ratio; SE: Standard error; *p < 0.05, **p < 0.01, ***p < 0.001

Similarly, AOR of discontinuing contraceptives due to husband disapproval was lower by 22% for young women compared to parenting adolescents. Multiple factors accounted for this disparity including living in a rural area (AOR = 1.37; p-value < 0.05), being ever married or cohabiting (AOR = 1.53; p-value < 0.05), as well as household wealth index in the middle (AOR = 0.75; p-value < 0.05) and richer categories (AOR = 0.70; p-value < 0.05). Several countries (Ethiopia, Kenya, Malawi, Namibia, Niger, Rwanda, Senegal, Zimbabwe, Tanzania, Burkina Faso and Zambia) had lower AORs of discontinuing contraceptives as compared to Angola, which also helped to explain why young women were less likely to discontinue contraceptives owing to husband disapproval than parenting adolescents. Wanting a more effective method was another reason for discontinuing contraceptives. The crude odds ratio of discontinuing contraceptives because of this reason was lower by 21% for young women compared to parenting adolescents. After taking into account the women’s individual characteristics and countries of residence, the disparity observed between young women and parenting adolescents, however, was eliminated.

From other regression results, we observed that AOR of discontinuing contraceptives among young women, compared to parenting adolescents, was higher by 1.34 times due to intention to become pregnant (i.e., wanted to get pregnant), higher by 1.29 times due to side effects or health concerns, lower by 30% due to access or availability issues, and lower by 24% due to infrequent sexual activity, or husband away or ill (analysis not shown). We found no disparities in the odds of contraceptive discontinuation between parenting adolescents and young women due to reasons related to inconvenience in using method; cost of method; fatalistic beliefs; difficulty getting pregnant, menopause or amenorrhea; and marital dissolution/divorced or widowed. Overall, the results indicate there were disparities in the odds ratios of discontinuing contraceptives between parenting adolescents and young women with respect to some but not all the reasons for discontinuing contraceptives.

## Discussion

We undertook a comparative analysis of reasons for discontinuing contraceptive use between childbearing adolescent girls (15–19-year-old) and young women (20–24-year-old) in selected sub-Saharan African countries. Examination of reasons for contraceptive discontinuation for the two groups (parenting adolescents and young women) shows that a greater proportion of parenting adolescents than young women discontinued contraceptives because of reasons such as pregnancy or method failure, husband disapproval, availability or access problems, desire to use more effective methods, inconvenience in using methods, infrequent sexual activity, cost of method, fatalistic beliefs, and marital dissolutions. Our multivariable analysis further highlighted lower odds of discontinuing contraceptive use due to many of the reasons established in the bivariate analysis (such as pregnancy or method failure and husband disapproval) among parenting young women relative to parenting adolescents. These results confirm our hypothesis that reasons for contraceptive discontinuation are different between the parenting adolescents and parenting young women. We conjecture some plausible reasons for these findings. Adolescents have poorer socioeconomic status than young women aged 20–24 [23, 26]. They are young and immature. Parenting adolescents may have lower household positions relative to 20–24-year-olds, which may lead to greater unequal power relation, and less ability to negotiate safer sex. Contraceptive discontinuation due to husband/partner disapproval may also be attributed to gender-based violence [12, 27].

Further, parenting adolescents are likely to have less education, and this may affect their knowledge about effective contraceptive use. Previous studies have emphasized the importance of education in increasing the knowledge of women about available contraceptives and methods choice [28, 29]. Parenting adolescents are likely to have less exposure which may also affect their contraceptive method choice.

The results also show that high proportion of parenting adolescents reported cost and access problem as some of the reasons for contraceptive discontinuation. These reasons may also be explained by their poor socioeconomic status and low household position. Studies have established a relationship between women’s low household position and poor maternal and child health outcomes [30, 31]. Although, we found that high percentages of both parenting adolescents and young women had poor socio-economic characteristics, our analysis demonstrates that parenting adolescents had poorer socio-economic status that make them highly vulnerable to poor sexual and reproductive health outcomes relative to young women. We also found lower proportion of modern contraceptive use among parenting adolescents relative to the young women. Our analysis confirms previous studies that suggest that adolescents face persistent socioeconomic inequalities that hamper progress towards the achievement of equity goals such as the Sustainable Development Goals and universal health coverage [4, 32].

Collectively, these results demonstrate that parenting adolescent girls are a high-risk public health group. Scholars have argued that first-time mothers and those who give birth before age 18 are a high-risk group for poor maternal health outcomes [2, 33, 34]. Izugbara et al. [2] reported that adolescent girls are a vulnerable maternal health group because they are less prepared for childbearing and motherhood; they use skilled healthcare providers less, and often have home delivery; and are more likely to have unsafe abortion.

## Limitations

While interpreting the findings of this study, some limitations should be borne in mind. First, our analysis was based on self-reported information. However, to mitigate this, data collectors received appropriate training on how to do proper interviewing and ensure the anonymity and confidentiality of solicited information in order to minimize bias. Second, due to the cross-sectional nature of the data, we could not explore a cause–effect relationship. Also, use of secondary data for the study constrains analysis to the available information in the datasets, thus limiting examination of other relevant variables such as socio-cultural values and practices regarding contraceptive use. In addition, the study did not include the random effects for the individual-level and country-level variables, and thus could not account for the unobservable variables that might influence contraceptive discontinuation across countries. Lastly, the study analysed data from some relatively old surveys for a few countries where more recent datasets are not available. This may pose some limitations due to the rapid transition in the contraceptive use landscape as a result of family planning interventions in some countries. Notwithstanding these drawbacks, the use of nationally representative samples for multiple countries is a key strength of the study. Also, the study fills an important gap in reproductive health literature on contraceptive discontinuations among parenting adolescents and young people in sub-Saharan Africa.

## Conclusion

The study established that there were disparities in the reasons for contraceptive discontinuation between parenting adolescents and young women, with adolescents demonstrating greater vulnerabilities and higher risks. The study demonstrate that parenting adolescents have lower socioeconomic status and are more likely to engage in risky sexual behaviour such as non-use of contraception compared to the parenting young women. This study concludes that special and considerable attention must be given to parenting adolescents in the efforts to achieve equity goal as part of the sustainable development goals and universal health coverage in sub-Saharan Africa.

## Data Availability

The datasets generated and analysed for the study are available on the Demographic and Health Survey (DHS) program website (dhsprogram.com).

## Abbreviations

AOR: Adjusted odds ratio
DHS: Demographic and Health Surveys
FP: Family planning
IPUMS: Integrated Public Use Microdata Series
IUD: Intra-uterine devices
SDGs: Sustainable Development Goals
SSA: Sub-Saharan Africa
